# Synopsis

**Published:** 1884-06

**Authors:** 


### PROCEEDINGS.

### Synopsis

OF THE MICHIGAN STATE DENTAL SOCIETY.

The twenty-eighth annual meeting of the Michigan State Dental Society was held in Detroit, beginning Wednesday evening, March 26, and continuing three days. About thirty members were present at the opening. The meeting was called to order by the President, Dr. W. H. Dorrance, of Ann Arbor.

The minutes of the last annual meeting were read and approved.

A programme for the future sessions was adopted. The State Board of Dental Examiners organized on the afternoon of the same day; of these there were present Dr. A. T. Metcalfe, of Kalamazoo, President; Dr. J. A. Robinson, of Jackson, Treasurer, and Dr. Geo. R. Thomas, of Detroit, Secretary.

The annual report of Secretary Thomas was read. In it he said: "It is well known to the members of the board what a struggle it has been, during the last fifteen years and more to make the State legislators recognize the fact that regarding the qualifications of dentists was necessary to protect the people from being consumed by charlatanism and quackery. Now that the struggle is over it is gratifying to note how little opposition there is manifested to the requirements of the law which went into effect September 8th last. Two days afterward as complete a list as was possible was prepared of the dentists of the State, and circulars mailed to them by me. Before the ninety days, the period for registration without examination had passed, 444 had registered. Thirty-four others had been added since the ninety days expired without examination."

Adjourned.

### SECOND DAY—MORNING SESSION.

The Society met at 9 o'clock. The President in the chair. After some routine business, Dr. Shattuck moved that a committee of three be appointed to prepare an amendment to the

Constitution, providing for associate and junior membership. This was adopted, and Drs. Clawson, Shattuck and Dorrance constitute the committee.

Dr. Geo. L. Field read the report of the University Visiting Committee. The report expressed great gratification with the condition of the dental college, the work being done, and the progress being made, and with the number of students in attendance.

The committee suggested the importance of an increase of the college term to nine months. The best interest of the students certainly demands such an extension. Further consideration of this subject was deferred.

The committee appointed last year to consider the matter of an inter-State association reported adversely to the proposition, and the report was adopted.

Dr. Land, of Detroit, read a short paper on full dentures. He took the position that plates were retained in the mouth more by the moisture than by air pressure. As soon as the mouth becomes adapted to the plate the direct pressure of the air ceases, and the denture is then maintained by its close contact, augmented by capillary attraction. There can be no direct pressure in the mouth without air space. The use of the chamber is two-fold; it forms a vacuum, and as a relief to the central portion of the platine arch. The former facilitates adaptation, the latter prevents the plate from riding upon the rigid portion of the dental arch.

The paper elicited considerable discussion. Dr. Field expressed great surprise at the views given in the paper. He criticised the use of the phrase air chamber; supposed vacuum was meant. The subject was further discussed by Drs. Clawson, Cleland, Long, and others, each one differing from Dr. Land.

Dr. E. Moore read a paper on "Best Methods and Material for Filling Teeth." The paper was interesting and instructive; advocating the selection of material adapted to the various cases. At the close of discussion upon this paper Dr. Watling moved that the calling of nitrous oxide gas and chloroform "vitalized air" is a misnomer, and calculated to deceive and mislead the public, and any member of this Association so advertising shall be guilty of a misdemeanor. Discussing this resolution Dr. Watling unhesitatingly pronounced such a course as unprofessional and quackish. He regretted to see that some of the older members of this Society are neglectful in this matter, and thus presenting a bad example to the younger members. In the discussion that followed, this substance, called "vitalized air" was shown to be a mixture of nitrous oxide gas and chloroform.

Dr. Long said that such imposition and deception, as is in this way practiced upon the people, should not be for one moment tolerated, at least by any member of this Society.

Adjourned.

### Afternoon Session.

The Society met according to adjournment for routine business. Dr. C. M. Cunningham and O. C. Jenkins were balloted for and admitted to membership.

Dr. Thomas; Chairman of the Executive Committee, read a number of advertisements of dentists in this State offering their services and material at greatly reduced rates. These advertisements, he stated, were in direct violation of the code of ethics of this Society. After the matter had been duly and fully considered the Executive Committee was instructed to act in the matter, as prescribed by the code.

On motion the Society went into an election of officers for the ensuing year, which resulted as follows:

President—Dr. J. Lathrop, of Detroit.

First Vice President—Dr. F. W. Clawson, of Detroit.

Second Vice President—Dr. E. C. Moore, of Detroit.

Secretary—Dr. J. B. McGregor, Port Huron.

Treasurer—Dr. H. K. Lathrop, Detroit.

Vacancy in Board of Censors—Dr. G. L. Field, of Detroit.

Adjourned.

### Evening Session.

Society was called to order at 7:30 p.m., after which, Dr. C. S. Case, of Ann Arbor, read a paper, or rather delivered a lecture, on "Oral Surgery." During the lecture the doctor illustrated his subject with many beautiful anatomical preparations, quite a number of which were prepared by himself; many interesting points were illustrated by these preparations. Especial reference was made to the antrum of Highmore, its position, size, and relations, were fully dwelt upon, and parts about it were fully discussed, their relations to one another, and to this cavity. He called attention to the naso-antral canal, its size, shape, direction, and the difficulties of effecting an entrance through it. The character of the walls, their relative thickness, the points at which they were most likely to be bulged, or ruptured, were clearly shown. He discussed somewhat at length the variety of diseases of this cavity, presenting the exact location of the palatine, artery, and nerve, and the Eustachian tube as well, and its susceptibility to injury in the engorgement of the antrum. The lecture received marked attention throughout. After this, Dr. Cowie, of Detroit, read a paper on the "Treatment of Deciduous Teeth." The paper mentioned among other things that the removal of bran from the flour in general use at the present day, destroyed to a great extent its bone-producing material, and that those who use such flour, are necessarily poorly nourished in this direction. The good quality of the teeth depends largely upon the general health and good nutrition. The paper insisted upon the greatest care in respect to cleanliness of the teeth of children, this should always be insisted upon. Foulness of teeth is often the occasion of general ill health. The greatest care and discrimination should be exercised in the removal of deciduous teeth to make room for the permanent set. This frequently causes the very trouble it is sought to obviate. Temporary teeth should be filled and kept in place as long as possible, unless special cause arises for further removal.

In discussion of the paper, Dr. Taft urged the necessity of thorough care of the teeth in infancy and childhood. This is to a large degree overlooked and neglected by a large proportion of the dental profession. The temporary teeth suffer from want of proper use; they suffer from impurities, and they suffer during times of illness, to which children are so frequently subject. The temporary teeth suffer much from open mouth while the child or person sleeps; the mouth should always be closed during sleep. Mothers or nurses should be careful to correct this bad habit in their children. Dr. Field asked how this could be done? The reply was that it is not easy to describe any particular method that will be applicable in all cases; but let the mother become thoroughly impressed with the danger of the habit, and she will usually find a way to meet the difficulty. A bandage may be used with very young children, and usually older ones may be so impressed with the importance of avoiding the habit that they will voluntarily, in a short time, correct it.

Dr. Harlan, of Chicago, remarked that he fully endorsed all that had been said. He very forcibly insisted upon the use of the tooth brush on the teeth of children, beginning very soon after they have teeth. The brush alone is not sufficient. A paste to accompany is prepared by the following formula. He has used it for many years; has found it efficient, and quite acceptable to children and adults as well:

| | | |
|---|---|---|
| Precipitated chalk | 2 | oz. |
| Powdered orris root | 2 | oz. |
| Powdered myrrh | 1 | dr. |
| Powered cuttle fish bone | ½ | oz. |
| White castile soap | ½ | oz. |
| Powdered white sugar | ½ | oz. |
| Glycerine and honey each | 1 | oz. |
| Rose pink | 8 | gr. |
| Oil of wintergreen | 20 | drops. |

This may be prepared by any good druggist, and for merely cleaning the teeth will render any other preparation unnecessary.

Dr. Thomas presented the report of the Executive Committee on the cases of violation of the code of ethics before referred to. He remarked that Drs. Stone and Essig, two of the infractors, had been before the committee. Dr. Stone had given reasons, which the committee considered satisfactory for the course he had pursued. He promised never again to transgress the code if he was forgiven for this offense. Dr. Essig asked time to consider: said he would report very soon. The Society accepted Dr. Stone's explanation, and therefore he retained his membership.

Dr. Thomas introduced Senator Houston, who had had charge of the Dental Bill during its progress in the Legislature. The Senator made some remarks in regard to the work in securing the passage of that bill; it had been put in his hands and he had done what he could for it, in the interest of the public. He desired to know more about the work of the dental college. He manifested a great interest in whatever pertains to the dental profession.

The thanks of the Association were extended to Senator Houston, after which the Society adjourned.

### THIRD DAY—Morning Session.

The Society met according to adjournment. After the transaction of some miscellaneous business the President called the attention of the Society to the very admirable report of the former day's proceedings of the Society, which appeared in the *Post and Tribune*. He commended very highly the completeness and accuracy of the report, and spoke of the great contrast between it and the reports of some other papers, in which it would seem that a great effort had been made to present untruthful, and even disrespectful, reports. Several other members gave expression to like sentiments. The Society expressed thanks for the report of the *Post and Tribune*.

Dr. Clawson moved that the Society instruct the officers and Executive Committee to arrange for a Dental Institute as a part of the work of the Association, in which all dentists of the State might become junior members.

Dr. Clawson traced the history of the Society during its existence, and commented on the many good ends it had attained. He believed that much more would have been done had all the dentists in the State been working together in one body. If the institute mentioned in the motion were organized it would have the effect of making the Association more powerful for good by assimilating the practice of all registered dentists. Junior members would be allowed to enter into the discussions of the Society, but not to vote.

Dr. Thomas seconded the resolution.

Dr. Robinson objected, because he believed all registered dentists could join the Association, if they desired, and he thought it hardly the thing to go out of the way to solicit them.

Other members strongly supported the resolution ; but it was amended, however, referring the matter to a committee of three, which consisted of Drs. Clawson, Dorrance, and Shattuck, to which the officers of the Society were added.

Dr. A. W. Harlan, of Chicago, was now introduced, and gave a very interesting lecture and clinic upon his method of the treatment of pyorrhœa alveolaris. The case upon which he operated was not a severe one, but one that required prompt treatment. The doctor operated in the case very thoroughly, and gave it the proper treatment. All were highly interested in his remarks and in the treatment.

Some discussion was now had upon the work of the Dental College. The statement was made that to complete the curriculum, as now presented, two terms, of six months each, is quite too short a time. The question now is whether they shall have three terms of six months each, or two terms of nine months each, as requisite for graduation. There is a great disposition on all hands for a lengthening of the term to nine months. It is throughout the university desired that the time of all the departments be made equal. The pressure upon the students in the effort to complete the curriculum in two six months' terms is so great that they desire to have longer time. In order to accomplish this extension it would be necessary to have a larger appropriation by the Legislature than heretofore. A committee should be appointed by this Society to give attention to this matter. The meeting be adjourned.

### Afternoon Session.

Association met at the usual time. A communication was read from Dr. I. Douglass, of Romeo, Michigan, in respect to the charges made against him of violation of the code of ethics. The doctor stated that he did not know what the charge against him was, but he was prepared to defend himself against any charge, and he would have appeared before the Society had he not been detained by the serious illness of his wife. The communication was referred to the Executive Committee.

On motion of Dr. Field, the President was authorized to appoint a committee of five to consider and act upon the report of the Visiting Committee in reference to extending the college term, and to take such action as they could for securing this end.

Dr. Rowley, of Niles, then read a paper on the treatment of "Exposed Pulps in Teeth." The paper took the position that whenever possible exposed pulps should be saved. He gave his method of treatment for securing this end.

In the discussion that followed the reading of the paper, various views were expressed as to the propriety of attempting to save exposed pulps as a general practice. It should always be attempted when there is any hope of success.

Dr. Robinson remarked that he always tried to save exposed pulps.

Dr. Thomas remarked that he saved his patient and himself much trouble by destroying exposed pulps as they are ordinarily presented.

Dr. W. H. Jackson believes that the effort to preserve the exposed pulps oftentimes involves the tooth so treated to liability of disease, and sometimes loss; and that, as a rule, it is safer to destroy the pulp.

Dr. Taft protested strongly against such practice; thought it was certainly wrong; would be sorry to have the impression go abroad that this Society endorsed the indiscriminate destruction of exposed pulps. In the hands of careful dentists the majority of exposed pulps, as presented ordinarily, may be preserved, and so the life of the tooth retained. A physician does not save all his patients. No one will deny that a tooth, with a live pulp, is much better than a tooth with a dead one. On motion the subject was passed.

Dr. H. C. Funk, of Cassopolis, was elected a member of the Association.

The Executive Committee, in reference to Dr. Douglass' communication, reported, and recommend that the matter be laid over for one year. The report was adopted.

A similar report was made in reference to the case of Dr. Finch, who was under the same charge as Dr. Douglass.

In reference to Dr. Essig's case the committee reported recommending delay. This was approved.

Dr. Land was heard in reference to the charge of violation of the code preferred against him. The charge being his unprofessional advertising. He said that if the code prohibited him from using the public press; in furthering his business, they could do him no higher honor than to expel him from the Association.

Dr. Case replied that he thought a dentist should be permitted to advertise anything he had for sale save his personal services.

The code of ethics was read, of which Dr. Land's advertisement is manifestly an infringement.

Dr. Jackson moved that Dr. Land be suspended from membership for one year.

Dr. Taft seconded the motion. He greatly regretted the course taken by Dr. Land; and hoped, after thinking the matter over, he would give up his mode of advertising.

Dr. Land said he preferred expulsion to temporary suspension. He believed there was an honorable course to pursue in professional advertising; that was to make the advertisements truthful. The code of ethics is wrong in that particular. Dr. Land was urged to withdraw his membership, rather than to submit to suspension; this he seemed quite willing to do.

Dr. Taft objected, remarking that the Society could not afford to permit Dr. Land to withdraw. No one can honorably withdraw, unless his record in the Society is a clear one. If the course pursued by him in advertising were generally adopted it would simply result in bringing the profession into disrepute and disgrace.

Dr. Land, upon further consideration, changed his mind, and promised to withdraw his advertisement for one year. This being done, the motion for suspension was laid upon the table.

The President-elect, Dr. J. Lathrop, was installed, and after returning thanks, the following committees were appointed.

Executive—Drs. Field, Watling, and Harris.

University Visiting Committee—Drs. Field, Moore, Lathrop, Cleland, and Davis.

Publishing Committee—Drs. Thomas, Dorrance, and McGregor.

Delegates to the American Dental Association—Drs. Peterson, McGregor, Case, Walton, Lathrop, Bean, and Shattuck.

On motion of Dr. E. C. Moore a vote of thanks was extended to Dr. A. W. Harlan for the interest he had added to the occasion. He was made an honorary member of the Association.

A vote of thanks was also extended to Hon. O. N. Case, member of the Michigan Legislature, for his assistance in the passage of bill. The Association then adjourned.

